# UV RESISTANCE LOCUS 8 From *Chrysanthemum morifolium* Ramat (CmUVR8) Plays Important Roles in UV-B Signal Transduction and UV-B-Induced Accumulation of Flavonoids

**DOI:** 10.3389/fpls.2018.00955

**Published:** 2018-07-04

**Authors:** Yanjun Yang, Xiuli Yang, Zhifang Jang, Zhehao Chen, Xiujun Ruo, Weiyang Jin, Ying Wu, Xiaojing Shi, Maojun Xu

**Affiliations:** ^1^Zhejiang Provincial Key Laboratory for Genetic Improvement and Quality Control of Medicinal Plants, Hangzhou Normal University, Hangzhou, China; ^2^Key Laboratory of Hangzhou City for Quality and Safety of Agricultural Products, College of Life and Environmental Sciences, Hangzhou Normal University, Hangzhou, China; ^3^College of Life and Environmental Sciences, Hangzhou Normal University, Hangzhou, China

**Keywords:** UV-B photoreceptor, CmUVR8, *Chrysanthemum morifolium* Ramat, flavonoids biosynthesis, COP1

## Abstract

UV Resistance Locus 8 (UVR8), an ultraviolet-B (UV-B; 280–315 nm) photoreceptor, participates in the regulation of various plant growth and developmental processes. UV-B radiation is an important factor enhancing the production of active components in medicinal plants. To-date, however, studies on UV-B photoreceptors have largely focused on *Arabidopsis*, and the functions of UVR8 in medicinal plants are still largely unknown. In the present study, a homolog of *Arabidopsis* UVR8, CmUVR8, was isolated from *Chrysanthemum morifolium* Ramat, and its structure and function were analyzed in detail. Protein sequence analysis showed that CmUVR8 contained nine conserved regulators of chromosome condensation 1 repeats, seven conserved bladed propellers, one C27 region, three “GWRHT” motifs and several crucial amino acid residues (such as 14 Trps and 2 Args), similar to AtUVR8. 3-D structural analysis of CmUVR8 indicated that its structure was similar to AtUVR8. Heterologous expression of *CmUVR8* could rescued the deficient phenotype of *uvr8-6*, a mutant of *UVR8* in *Arabidopsis*, indicating the role of CmUVR8 in the regulation of hypocotyl elongation and *HY5* gene expression under UV-B irradiation. Moreover, CmUVR8 regulates UV-B-induced expression of four flavonoids biosynthesis-related genes and the UV-B-induced accumulation of flavonoids. Furthermore, the interaction between CmUVR8 and CmCOP1 were confirmed using a yeast two-hybrid assay. These results indicated that CmUVR8 plays important roles in UV-B signal transduction and the UV-B-induced accumulation of flavonoids, as a counterpart of AtUVR8.

## Introduction

Light is important in regulating plant growth and development. Light acts not only as the primary source of energy through photosynthesis, but also conducts intricate cellular signaling pathways through a series of photoreceptors (Sullivan and Deng, 2003). Higher plants are now known to possess five categories of photoreceptors, including red/far-red light receptor phytochromes (Quail et al., 1995), phototropins (Briggs and Huala, 1999), cryptochromes (Cashmore et al., 1999), Zeitlupe family members (Chen et al., 2004), and the Ultraviolet-B (UV-B) photoreceptor UV RESPONSE LOCUS 8 (UVR8) (Rizzini et al., 2011).

UV-B radiation (280–315 nm) is an intrinsic part of sunlight. Although the proportion of UV-B in total light energy reaching the earth’s surface is less than 0.5%, UV-B has important regulatory effects on the growth and developmental processes of plants. UV-B controls various photomorphogenic responses, including hypocotyl growth inhibition, stomatal opening, phototropic curvature, cotyledon expansion, and anthocyanins and flavonoids biosynthesis (Kim et al., 1998; Boccalandro et al., 2001; Suesslin and Frohnmeyer, 2003).

Plants perceive and respond to UV-B radiation through a unique UV-B photoreceptor. To date, UVR8 is the only defined UV-B photoreceptor (Rizzini et al., 2011). UVR8, which was identified in a screen for *Arabidopsis* mutants hypersensitive to UV-B exposure, and has sequence similarity to the proteins of the RCC1 family (Kliebenstein et al., 2002). *Arabidopsis* UVR8 protein contains a well-conserved seven-bladed-propeller core, a short N-terminal extension and a flexible C-terminal region with about 60 amino acids that contains a C27 region (Jenkins, 2014). The UVR8 protein is localized in both the nucleus and cytoplasm and rapidly accumulates in the nucleus under UV-B irradiation (Brown et al., 2005; Kaiserli and Jenkins, 2007). In the nucleus, UVR8 binds to the chromatin of several UV-B responsive genes, including the *ELONGATED HYPOCOTYL 5* (*HY5*) promoter region, probably through interaction with histones (Brown et al., 2005; Cloix and Jenkins, 2008).

Unlike other photoreceptors that contain external cofactors such as chromophores, UVR8 does not have a prosthetic chromophore. Instead, the protein sequence of UVR8 contains 14 well-conserved tryptophans (Trps) as chromophores in photoreception (Wu et al., 2012; Jenkins, 2014). Two of these Trps, Trp 233 and Trp 285, are collectively served as the UV-B chromophore (Christie et al., 2012; Wu et al., 2012). Studies on the crystal structure of the UVR8 domain revealed that UVR8 forms a stable dimer by a number of salt bridges without UV-B irradiation. However, UV-B radiation of the excitable Trps leads to the effective transfer of an excited electron from the excitonically coupled Trp pyramid to adjacent Args, leading to charge neutralization and subsequent dimer dissociation (Christie et al., 2012; Wu et al., 2012). As a homology of RCC1 proteins, UVR8 was regarded to interact with chromatin at the loci of some target genes through histone binding, thus regulating the expression of a series of UV-B induced downstream genes (Cloix and Jenkins, 2008). However, a recent study showed that UVR8 binding to chromatin could not be reproduced through intensive diverse experiments (Binkert et al., 2016).

Although the intrinsic signal transduction pathway of UVR8 is not well understood, it is generally acknowledged that COP1 (CONSTITUTIVELY PHOTOMORPHOGENIC 1) and HY5, two important factors in the light signaling pathway, play major roles in photomorphogenesis under UV-B exposure. In plants, COP1, an E3 ubiquitin ligase, is best known for its function as a photomorphogenesis repressor (Yi and Deng, 2005). However, COP1 also has a positive function in UV-B induced light signaling (Boccalandro et al., 2004; Oravecz et al., 2006). In *Arabidopsis*, UVR8 interacts with the WD40-repeat domain of COP1 in a UV-B-dependent way, and this interaction is closely related to downstream UV-B specific responses (Oravecz et al., 2006; Favory et al., 2009; Cloix et al., 2012). The basic leucine-zipper transcription factor HY5 is an important regulator of photomorphogenic responses that are mediated by various photoreceptors (Jiao et al., 2007; Lee et al., 2007). Both UVR8 and COP1 are involved in the UV-B-mediated transcriptional activation of *HY5* (Brown et al., 2005; Oravecz et al., 2006). Mutation of *HY5* inhibits the expression of a series of UV-B-induced downstream genes (Brown et al., 2005; Oravecz et al., 2006).

Although UV-B and its photoreceptor UVR8 play important roles in regulating plant growth and development, (Kliebenstein et al., 2002; Brown et al., 2005; Favory et al., 2009; Wargent et al., 2009; Mao et al., 2015; Zhao et al., 2016), studies of UV-B photoreceptors have mainly focused on the model plant *Arabidopsis*. UV-B irradiation is an important factor enhancing the production of defense-related secondary metabolites in plants (Julkunen-Tiitto et al., 2005). Many secondary metabolites, such as flavonoids, alkaloids, and lignin, are active components of medicinal plants. Although UV-B induced accumulation of active components in medicinal plants is widely approved (Zhang and Bjorn, 2009), there is still no research about the UV-B photoreceptor and UV-B signal transduction pathway in medicinal plants. To improve our understanding of the regulation mechanism for UV-B-induced accumulation of active components in medicinal plants, it is important an essential to pursue research on UV-B photoreceptor and UV-B signaling pathway in medicinal plants are important and essential.

*Chrysanthemum morifolium* Ramat (*C. morifolium*) is a traditional Chinese medicine in which flavonoids are the main bioactive compounds. Since the co-relation between UV-B and UV-B induced secondary metabolites is still largely unknown in medicinal plants, study on UV-B transduction and UV-B-induced accumulation of flavonoids biosynthesis in *C. morifolium* will provide a typical model. In the present study, a UV-B light photoreceptor gene, *CmUVR8*, was isolated from *C. morifolium*, and structural and evolutionary relationships between CmUVR8 and the UVR8s from other species were also analyzed. Functional complementation assays using an *Arabidopsis uvr8* mutant were conducted to confirm the role of CmUVR8 in regulating hypocotyl growth, gene expression and flavonoids accumulation in plants. Additionally, the interaction between CmUVR8 and CmCOP1 was confirmed to investigate the functional mechanism of CmUVR8. Our results shed new light on the important roles of the UV-B photoreceptor and its signal transduction pathway in medicinal plants, particularly in *C. morifolium*.

## Materials and Methods

### Plants, Growth Conditions, and UV-B Treatments

*Arabidopsis thaliana* seeds, including wild-type (accession Col-0) and a T-DNA insertion line *uvr8-6*, were used in our study (Favory et al., 2009). Seeds were germinated on 1/2 Murashige and Skoog medium and transferred to a culture room under a long-day (light/dark: 16/8 h) condition at 22°C.

For the UV-B treatments, *Arabidopsis* seedlings were exposed to successive low fluency rate white light (Osram L18W/30 tubes, 20 μmolm^-2^s^-1^) supplemented with narrowband UV-B (Q-Lab narrowband UV-B tubes, 1.5μmolm^-2^s^-1^). The UV-B light was adjusted by using 3-mm transmission cutoff filters, containing 305 nm cutoff and 345 nm cutoff filters (WG305 and WG345; Schott, Mainz, Germany; Oravecz et al., 2006; Favory et al., 2009).

### Isolation of Full-Length cDNA of *CmUVR8*

To obtain the full-length cDNA of CmUVR8, CmUVR8-F1, and CmUVR8-R1 primers were designed based on our previous transcriptome data of *C. morifolium*. Leaves of 3-month-old *C. morifolium* seedlings were harvested, frozen immediately in liquid N_2_, and then ground using a mortar and pestle. Total RNAs were isolated using the RNApure Plant Kit (CoWin Biotech, Beijing, China) according to manufacturer instructions. RNAs were reverse transcribed into cDNA using a HiFiScript cDNA Synthesis Kit (CoWin Biotech, Beijing, China). Full-length cDNA of CmUVR8 were obtained by RT-PCR. The PCR primer sequences are all listed in Supplementary Table S1.

Multiple alignments of amino acid sequences were performed with Clustal Omega sequence alignment, and the phylogenetic tree was constructed with MEGA 7 software using the neighbor-joining method.

### Real-Time Quantitative and Semi-Quantitative RT-PCR Analysis

For tissue-specific gene expression analysis, samples of shoots, roots, stems, and flowers were collected from 8-month-old adult *C. morifolium* of the cultivar ‘xiaoyangju’ planted in natural conditions in Tongxiang, China.

For qRT-PCR, a pair of specific primers (qCmUVR8-F and qCmUVR8-R) were designed. qRT-PCR was performed using a MyiQ Single Color Real-time PCR system (Bio-Rad, Hercules, CA, United States) with a SYBR Green Master Mix (CoWin Biotech, Beijing, China), following manufacturer instructions. The procedures for PCR were as follows: 95°C for 10 min; 95°C for 15 s, 40 cycles; 60°C for 60 s. For tissue-specific gene expression analysis, the *CmTubulin* gene was used as the internal reference. Relative fold changes were calculated basing on comparative cycle threshold (2^-ΔΔC_t_^) values. For expression analysis of UV-B-induced genes, the *AtActin 2* gene was used as the internal reference. All the experiments were repeated three times. Semi-quantitative RT-PCR analysis was performed using qCmUVR8-F and qCmUVR8-R as primers.

### Generation of CmUVR8-Transgenic *Arabidopsis* Plants

CmUVR8 ORF was amplified using primers of CmUVR8-F2 and CmUVR8-R2 and cloned into the vector pCAMBIA 13011 under the control of the 35S promoter. The construct was transformed into *Arabidopsis* plants by the *Agrobacterium tumefaciens* strain *GV3101*. *Arabidopsis* was transformed by the floral dip method (Clough and Bent, 1998). T3 plants were used for subsequent analysis.

### Hypocotyl Measurements

Seeds of four *Arabidopsis* lines were germinated in agar plates. The plates were placed vertically to make the seedlings straight. Hypocotyl lengths of 4-day-old seedlings grown with or without UV-B light were measured (30 seedlings each group).

### Measurement of Flavonoid Concentration

Sample extraction and HPLC-MS analysis of flavonoids were performed as described before with light modification (Tohge et al., 2005). Frozen samples were homogenized in a 50 μl extraction solvent with methanol: acetate: H_2_O = 9:1:10 per 1 mg (dry weight) of samples by pestle and mortar at 37°C for 30 min. After centrifugation at 12,000 × *g* for 5 min, cell debris was scraped and the remaining extracts were centrifuged again. Then, the supernatant was filtered by filter membrane (0.25 μm) and 10 μl of the resulting supernatant was loaded to a HPLC-MS system comprising a waters HPLC e2695 series (Waters, United States) and a Bruker maXis UHR-TOF mass spectrometer (maXis UHR-TOF, Bruker Daltonics, Bremen, Germany). HPLC was performed on an XBridge C18 (Φ4.6 mm × 250 mm) column, and the flow rate was 0.5 ml/min. The elution solutions were as follows: solvent A [CH_3_CN:H_2_O:TFA (10:90:0.1)]; and solvent B [CH_3_CN:H_2_O:TFA (90:10:0.1)]. The elution profile was as follows: 100% of A for 0 min, 70% of A for 30 min, 0% of A for 32 min, 0% of A for 33 min, and 100% of A for 35 min. UV-visible absorption between 190 and 510 nm was examined using PDA. Flavonoids were determined at a wavelength of 320 nm. The MS analysis was performed using a Bruker maXis UHR-TOF mass spectrometer (maXis UHR-TOF, Bruker Daltonics, Bremen, Germany). Nitrogen was treated as sheath gas for the positive-ion mode, flow rate and voltage of 180°C, 6 L/min and 4.5 kV, respectively. Atomizing gas pressure was 0.4 bar. Full mass spectra scan was acquired from 200 to 1500 D.

### Yeast Two-Hybrid Assays

Yeast two-hybrid analysis was performed using the Matchmaker GAL4 Two-Hybrid System 3 (Clontech, Mountain View, CA, United States) following the manufacturer’s protocol. ORF of the *CmUVR8* gene was cloned into pGBKT7 vector using primers YCmUVR8-F and YCmUVR8-R, and transformed into yeast Y187 using the lithium acetate-based transformation method. ORF of CmCOP1 was cloned into pGADT7 using primers YCmCOP1-F and YCmCOP1-R which were also designed based on our previous transcriptome data of *C. morifolium*, and transformed into yeast AH109.

Mated strains were spread on SD media lacking Leu (SD-Leu), Trp (SD-Trp), Leu and Trp (SD-Leu/-Trp) or SD media, lacking Ade, His, Leu and Trp, with the chromogenic substrate X-α-Gal (5-bromo-4-chloro-3-indolyl-α-D-galactopyranoside) (SD-Ade/-His/-Leu/-Trp/X-α-Gal) for interaction selection. Positive control (pGBKT7-53 and pGADT7-T) and negative control (pGBKT7-Lam and pGADT7-T) were used. Yeast cells were irradiated using a UV-B narrowband (20 h, 1.5 μmolm^-2^s^-2^) (Yin et al., 2015).

## Results

### Cloning of the Full-Length cDNA of CmUVR8

According to the sequence obtained from our previous transcriptome data, primers were designed to isolate the full-length sequences of *CmUVR8* from the leaves of *C. morifolium*. After sequencing, the full-length cDNA sequence of *CmUVR8* had a length of 1521 bp, which contains an ORF with a length of 1,320 bp. The deduced CmUVR8 protein was comprised of 439 amino acids with 47.6 kDa and an isoelectric point of 5.49.

### Analysis of the CmUVR8 Protein Sequence

**FIGURE 1:**
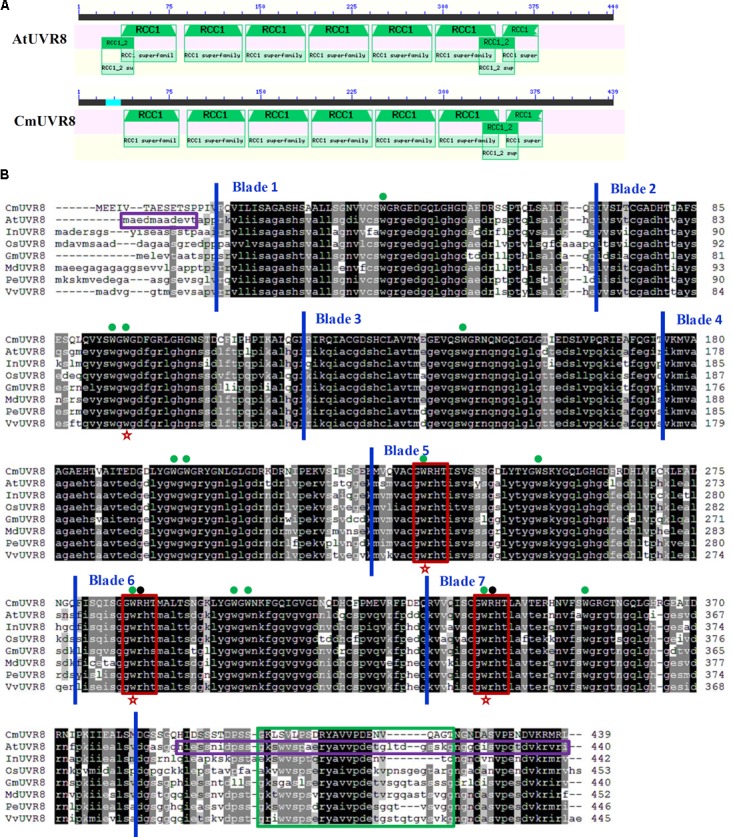
Domains analysis of the AtUVR8 and CmUVR8 proteins. **(A)** RCC1 domain comparison of AtUVR8 and CmUVR8. Amino acid sequences of UVR8s were analyzed in the National Center for Biotechnology Information (NCBI) database using CD-search software (http://structure.ncbi.nlm.nih.gov/Structure/cdd/wrpsb.cgi). RCC1: pfam00415; RCC1_2: pfam13540. **(B)** Amino acid sequence alignment of CmUVR8 with orthologs from various species. The alignment was performed using the Clustal Omega on line. Black boxes indicate amino acid residues that are >80% conserved, and gray boxes indicate amino acids that are >50% conserved. Blue vertical lines define the seven blades in CmUVR8. Green dots above the sequences indicate the 14 conserved Trp residues. The red stars under the sequences indicate the triad Trps (W233, W285, and W337, positions 235, 287, 339 in the CmUVR8 protein sequence) that form a pyramid arrangement with W94 (positions 96 in CmUVR8) on the adjacent monomer. Black dots above the sequences indicate the two Arg residues (R286 and R338, positions 288 and 340 in CmUVR8) which are important to maintain the dimer of AtUVR8. Green frames indicate the C27 region, and red frames indicate the three conserved “GWRHT” motifs. Purple frames indicate the missing residues in the AtUVR8 crystal structure. Cm, *Chrysanthemum morifolium*; At, *Arabidopsis thaliana* (AED97805); In, *Ipomoea nil* (XP_019200437); Os, *Oryza sativa* (XP_015636973); Gm, *Glycine max* (XP_003526878); Md, *Malus domestica* (AMZ80131); Pe, *Populus euphratica* (AKJ54489); Vv, *Vitis vinifera* (CBI21608).

In *Arabidopsis*, UVR8 has sequence and predicted structural similarity to human REGULATOR OF CHROMATIN CONDENSATION 1 (RCC1) (Kliebenstein et al., 2002). To detect whether the *UVR8* gene from *C. morifolium* has sequence similarity to human RCC1 as *AtUVR8*, these two sequences were both analyzed using CD-search. Sequence analysis revealed that, similar to AtUVR8, the CmUVR8 protein sequence contained seven classical conserved RCC1 repeats (**Figure 1A**). However, it contained only one domain belonging to RCC1_2 superfamilies, in contrast with the two domains in AtUVR8 protein (**Figure 1A**).

Previous studies demonstrated that UVR8 is a conserved protein that existed widely in various plant species (Fernaandez et al., 2016). Unlike other known photoreceptors, specific Trps rather than a prosthetic chromophore were used for UVR8 to absorb light during UV-B photoreception (Jenkins, 2014). To confirm these conserved domains and residues, the CmUVR8 protein sequence was compared with the UVR8s from other seven representative plants (**Figure 1B**). Like AtUVR8, CmUVR8 also contained a seven-bladed-propeller core, a short N-terminal extension and a flexible C-terminal region. Besides, CmUVR8 also has 14 highly conserved Trps, like AtUVR8 (**Figure 1B**).

In AtUVR8, the two pyramidal arrangements per dimmer which are formed by a Trp on one monomer and a triad of Trps on another are key to maintain the dimmer (Christie et al., 2012; Wu et al., 2012). In addition, two Args (R286 and R338) were also important for maintaining the dimmer (Christie et al., 2012; Wu et al., 2012). The four essential Trps and the two important Args are well conserved in CmUVR8, which has Trps (W96, W235, W287, and W339) and Args (R288 and R340) in the amino acid sequence (**Figure 1B**).

In *Arabidopsis*, the three Gly-Trp-Arg-His-Thr in blades 5-7 were important to generate the triad Tryps. They were also conserved in the CmUVR8 sequence, and also distributed in 5–7 blades like in AtUVR8 (**Figure 1B**). In addition, the C27 region, which is required for UVR8 activity, was also largely conserved in the CmUVR8 sequence (**Figure 1B**).

### Structural and Phylogenetic Analysis of CmUVR8 Protein

**FIGURE 2:**
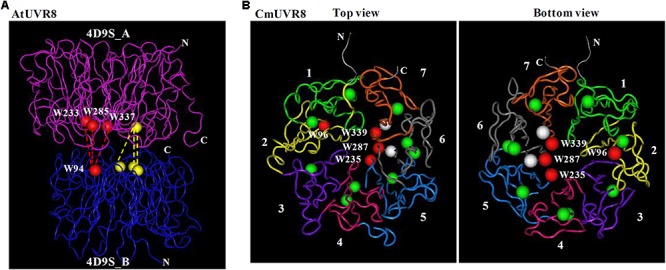
Structural analysis of CmUVR8 protein. **(A)** Dimer structure of the AtUVR8. Red and yellow balls represent important Trps in the two pyramids between the two monomers. **(B)** Predicted 3D structure of the CmUVR8 protein. The 3D structure of CmUVR8 is predicted in Phyre 2 (http://www.sbg.bio.ic.ac.uk/phyre2/html/page.cgi?id=index) and drawn by Cn3D software. The seven blades of CmUVR8 are represented by seven different colors and indicated by number 1–7. Red and green balls indicate the 14 conserved Trps with the red balls representing the four important Trps in the pyramids (W96, W235, W287, and W339). White balls indicate the two Args (R288 and R340) that are important to maintain the dimer structure.

Under UV-B irradiation, UVR8 undergoes a quick switch from homodimer to a monomer that triggers a pathway for UV-B protection (Christie et al., 2012; Wu et al., 2012). Crystallographic and solution structures of the UVR8 homodimer reveal that the concave surfaces of two UVR8 dimers assemble face to face, stitched together by a salt-bridge network (**Figure 2A**) (Christie et al., 2012; Wu et al., 2012). To investigate the structure of CmUVR8 in detail, the 3D structure of CmUVR8 was predicted and analyzed. Comparisons between CmUVR8 and AtUVR8 was conducted using the crystal structure of AtUVR8 (PDB: 4D9S_A) as a model. The result suggested that the CmUVR8 protein also possessed a seven-bladed-propeller (**Figure 2B**). Furthermore, the four important Trps and two important Args are all conserved in CmUVR8 (**Figure 2B**).

**FIGURE 3:**
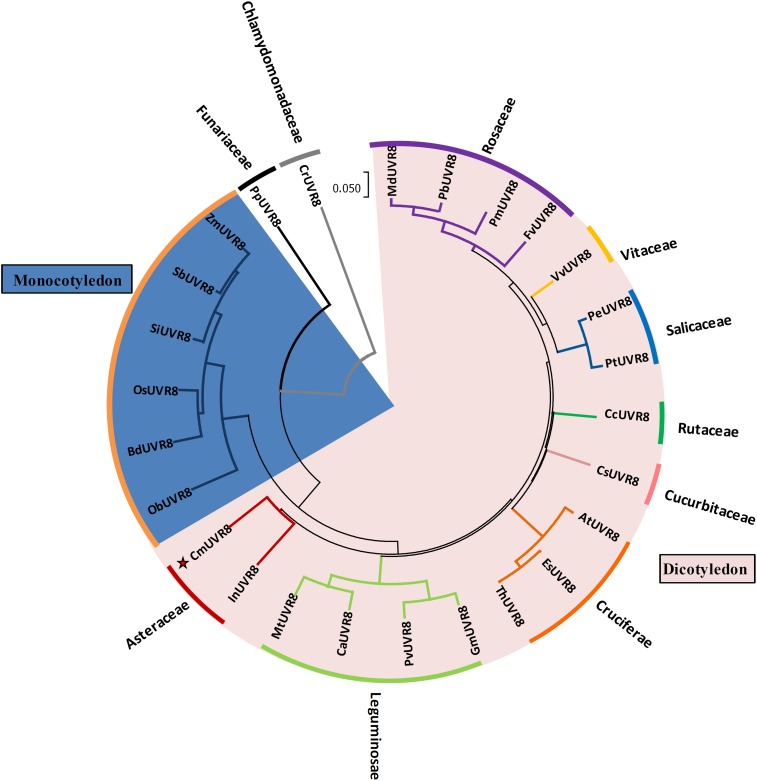
Phylogenetic tree of UVR8 proteins in plants. Amino acid sequences of UVR8s from 26 plant species were searched and obtained from the NCBI database using the BLASTP method. The alignment was carried out using Clustal Omega, and the phylogenetic tree was constructed using the neighbor-joining method in the MEGA 7 software (version 7.1). The bootstrap value was 1,000 replicates. The scale bar represents 0.05 substitutions per sequence position. CmUVR8 is marked by a red star. At, *Arabidopsis thaliana* (AED97805); Bd, *Brachypodium distachyon* (XP 003579788); Ca, *Cicer arietinum* (XP_004502975); Cc, *Citrus clementina* (XP_006432102); Cr, *Chlamydomonas reinhardtii* (AKS29686); Cs, *Cucumis sativus* (XP_011653892); Es, *Eutrema salsugineum* (XP_006394235); Fv, *Fragaria vesca* (XP_004288153); Gm, *Glycine max* (XP_003526878); In, *Ipomoea nil* (XP_019200437); Md, *Malus domestica* (AMZ80131); Mt, *Medicago truncatula* (XP_003602664); Ob, *Oryza brachyantha* (XP 006652309); Os, *Oryza sativa* (NP 001052849); Pb, *Pyrus bretschneideri* (XP_009355901); Pe, *Populus euphratica* (AKJ54489); Pm, *Prunus mume* (XP_008227523); Pp, *Physcomitrella patens* (XP_001778783); Pt, *Populus trichocarpa* (XP_002309939); Pv, *Phaseolus vulgaris* (XP_007137765); Sb, *Sorghum bicolor* (XP 002446509); Si, *Setaria italica* (XP 004975662); Th, *Thellungiella halophila* (BAJ33982); Vv, *Vitis vinifera* (CBI21608); Zm, *Zea mays* (NP 001141147).

To investigate the phylogenetic relationships between CmUVR8 and its homologs from other plant species, a phylogenetic tree was built. CmUVR8 amino acid sequences were aligned with sequences of UVR8 homologs from several other plants (25 species), including19 dicotyledonous species from six genera, seven monocotyledonous species, one moss, and one chlamydomonas. The phylogenetic tree showed that UVR8 from different species are well conserved (**Figure 3**). As expected, CmUVR8 grouped in the dicotyledon UVR8 clade. CmUVR8 and InUVR8 (*Ipomoea nil*) were classed into the same clade, indicating that they were most closed (**Figure 3**). Relatively, CmUVR8 was distantly closed to the UVR8s from monocotyledonous plants, moss and chlamydomonas. These results showed that there is a significant boundary among the different UVR8 proteins from dicotyledonous and monocotyledonous species (**Figure 3**).

### Tissue-Specific Expression Pattern of *CmUVR8* Gene

**FIGURE 4:**
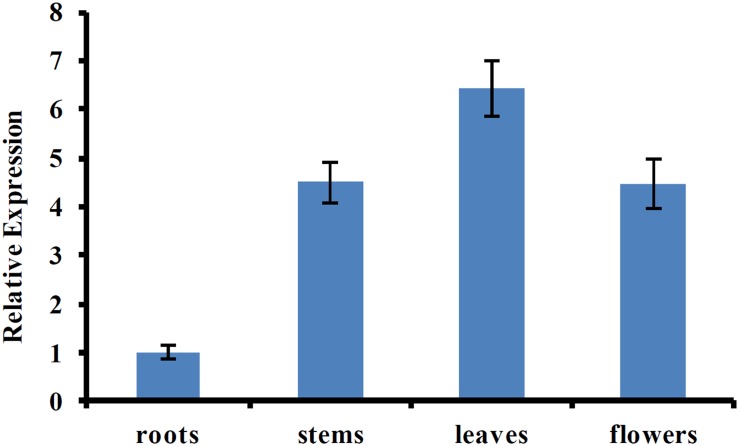
Tissue-specific expression pattern of *CmUVR8* gene. Expression pattern of *CmUVR8* gene in four indicated tissues (roots, stems, leaves, and flowers) was analyzed by real-time RT-PCR. The *CmTubulin* gene was performed as an internal control. The data were analyzed by three biological replicates, and standard deviations were shown with error bars.

Previous studies showed that AtUVR8 was a ubiquitously expressed gene, and this expression pattern allows plants to immediately respond to UV-B exposure (Favory et al., 2009; Rizzini et al., 2011). To determine the tissue-specific expression pattern of CmUVR8, qRT-PCR was performed using the samples from different tissues, including roots, stems, leaves, and flowers. The result showed that CmUVR8 was expressed in all the four tissues, but the expression levels varied among different tissues (**Figure 4**). CmUVR8 expressed highest in leaves, followed by flowers, and expressed relatively weakly in roots (**Figure 4**).

### Functional Complementation Study of CmUVR8 in *Atuvr8* Mutant

**FIGURE 5:**
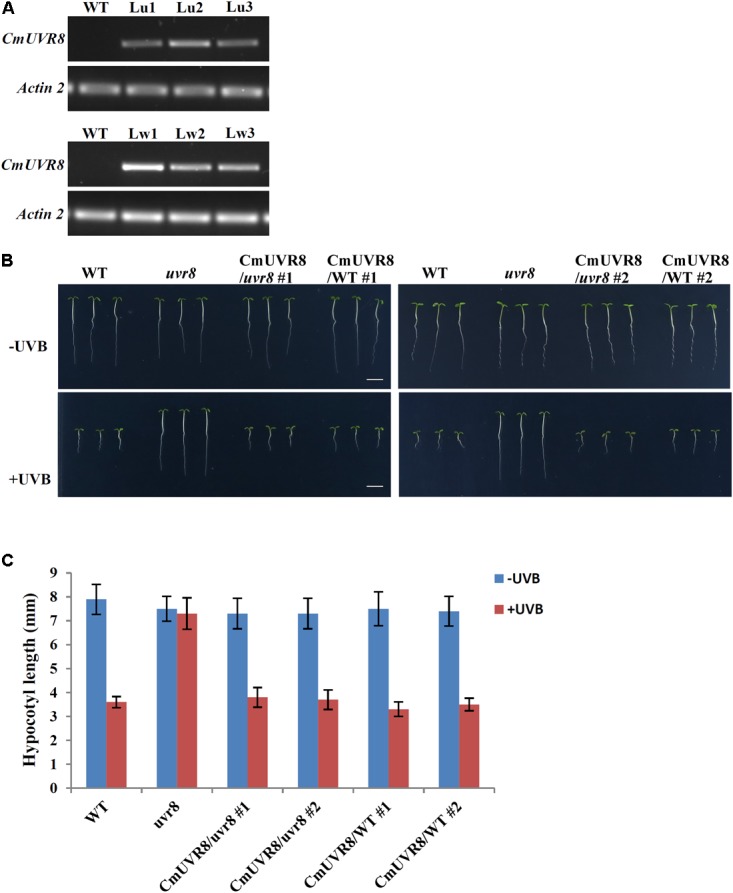
Phenotypes of WT, *uvr8* mutant, *CmUVR8*-transgenic *uvr8* mutant, and *CmUVR8*-transgenic WT plants. **(A)** Expression levels of CmUVR8 in 4-day-old seedlings of the four lines. Lu1, Lu2, Lu3, three transgenic lines in the *uvr8* mutant background; Lw1, Lw2, Lw3, three transgenic lines in WT background. **(B)** Phenotypes of the four lines (WT, *uvr8* Lu2 and Lw1) grown under white light with or without UV-B irradiation. Scale bar represents 5 mm. **(C)** Comparison of the hypocotyl length of 4-day-old seedlings of the four lines under white light with or without UV-B irradiation. Error bars indicate SD (*n* > 30).

AtUVR8 inhibits hypocotyl growth and activates gene expression, such as *HY5* and *CHS* (*Chalcone synthase*) in the process of UV-B photomorphogenesis (Favory et al., 2009). To reveal the functions of CmUVR8, functional complementation assay using an *uvr8-6* mutant was conducted (Favory et al., 2009). CmUVR8 was introduced into the pCAMBIA13011 vector driven by a 35S promoter. The 35S::CmUVR8 construction was transformed into both the wild-type (WT; Col-0 ecotype) and *uvr8* mutant. Transgenic expression levels were determined using RT-PCR (**Figure 5A**). The results showed that CmUVR8 was successfully transformed into *Arabidopsis* WT and *uvr8* mutants.

### CmUVR8 Inhibits Hypocotyl Elongation in *Arabidopsis*

To determine the role of CmUVR8 in hypocotyl growth, hypocotyl growth inhibition induced by UV-B was investigated in the four different *Arabidopsis* plants (WT, *uvr8* mutant, *uvr8* mutant transformed with CmUVR8, and CmUVR8 overexpressing lines). Two transgenic lines for *uvr8* mutant transformed with CmUVR8 and CmUVR8 overexpressing lines were used for the subsequent experiments. As shown in **Figure 5B**, there was no obvious difference in hypocotyl length of the four lines when grown under white light. However, when UV-B light was applied, the *uvr8* mutant displayed reduced inhibition of hypocotyl growth, and this reduction could be restored by the *CmUVR8* transformation (**Figures 5B,C**). Our data verified that CmUVR8 plays a role in hypocotyl elongation in plants under UV-B irradiation.

### CmUVR8 Promotes the Expressions of *HY5* and Flavonoid Biosynthesis Related Genes in *Arabidopsis*

**FIGURE 6:**
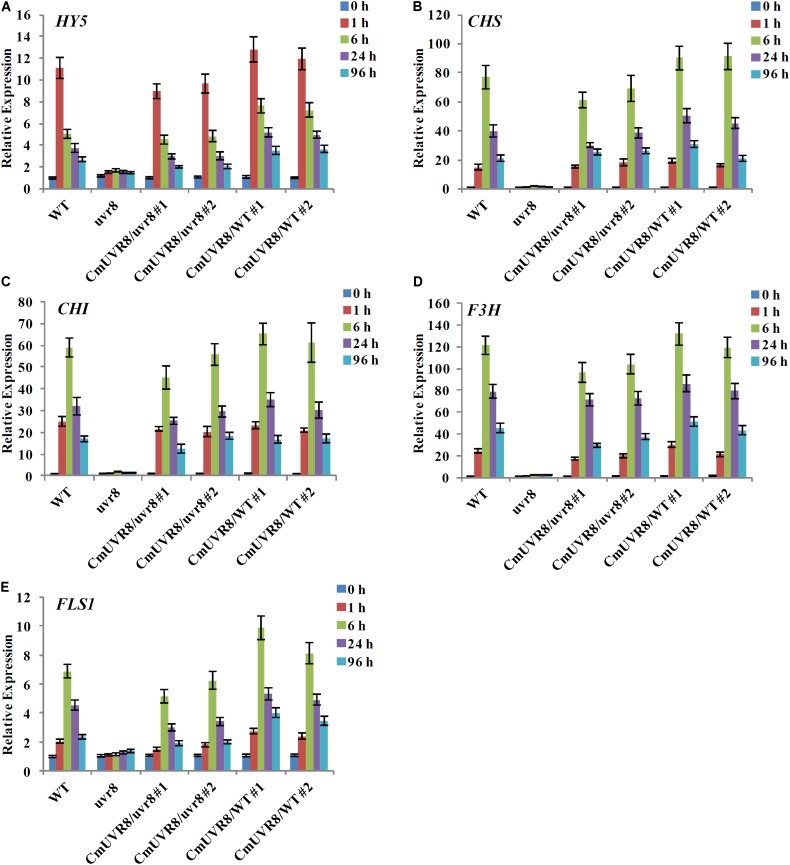
UV-B-dependent induction of *HY5* gene and four flavonoid biosynthesis genes in WT, *uvr8* mutant and the two transgenic lines. The expression level of *HY5* **(A)**, *CHS* **(B)**, *CHI* **(C)**, *F3H* **(D)**, and *FLS3* **(E)** genes in four lines with different durations of UV-B irradiation was analyzed by real-time RT-PCR. The data was analyzed by three biological replicates, and standard deviations were shown with error bars.

In *Arabidopsis*, *uvr8* mutants exhibited a lack of UV-B-induced gene expression, including *HY5* and *CHS*, and a lack of UV-induced flavonoids accumulation (Favory et al., 2009). To further investigate the role of CmUVR8 in photomorphogenesis, the expression level of *HY5* and four important flavonoid biosynthesis genes, including *CHS*, *CHI* (*Chalcone isomerase*), *F3H* (*Flavonoid 3-hydroxylase*), and *FLS1* (*Flavonol synthase 1*), were detected in the four types of *Arabidopsis* lines. *Arabidopsis* seedlings of the four lines were planted under white light supplemented with UV-B irradiation for 96 h, and qRT-PCR was used to detect the expression levels of HY5 and flavonoid biosynthesis-related genes at different time points. Under UV-B exposure, all five genes in *Arabidopsis* Col-0 seedlings exhibited apparently UV-B-induced expressions, while all these genes exhibited a lack of UV-B-induced expressions in the *uvr8* mutants. Transformation of the *uvr8* mutant with CmUVR8 restored UV-B-induced gene expression (**Figures 6A–E**). Furthermore, CmUVR8-overexpressing lines exhibited the highest levels of gene expression, similar to *Arabidopsis* lines overexpressing *AtUVR8* (**Figures 6A–E**) (Favory et al., 2009). Our results showed that CmUVR8 played important roles in the promotion of the expression of *HY5* and flavonoid biosynthesis related genes.

### CmUVR8 Increases the Accumulation of Flavonoids in *Arabidopsis*

**FIGURE 7:**
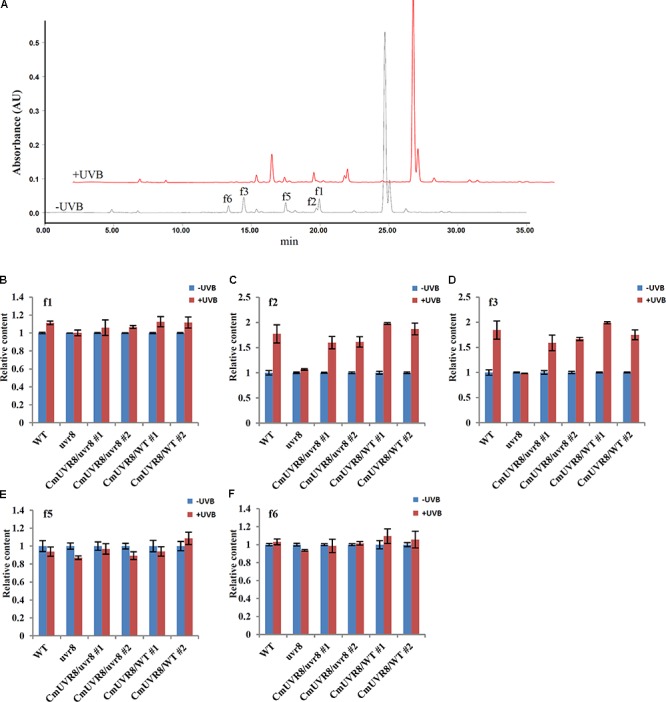
UV-B-induced flavonoids accumulation of WT, *uvr8* mutant, and two transgenic lines. **(A)** HPLC fingerprints of the *Arabidopsis* seedlings treated with (+UVB) or without UV-B (–UVB). Absorption was at the wavelength of 360 nm. **(B–F)** Relative flavonoids content of WT, *uvr8* mutant, and two transgenic lines treated with or without UV-B radiation. f1: Kaempferol 3-*O*-rhamnoside 7-*O*-rhamnoside, f2: Kaempferol 3-*O*-glucoside 7-*O*-rhamnoside, f3: Kaempferol 3-*O*-[6”-*O*-(rhamnosyl) glucoside] 7-*O*-rhamnoside, f5: Quercetin 3-*O*-glucoside 7-*O*-rhamnoside, f6: Quercetin 3-*O*-[6”-*O*-(rhamnosyl) glucoside] 7-*O*-rhamnoside. The data were analyzed by three biological replicates, and standard deviations were shown with error bars.

Previous studies showed that the content of flavonoids was low in the *uvr8* mutants and flavonoids accumulated significantly in *AtUVR8* overexpressing lines grown under white light supplemented with UV-B light (Favory et al., 2009). To further study the effects of CmUVR8 on flavonoids biosynthesis, 3-week-old *Arabidopsis* seedlings were exposed under UV-B radiation for 24 h, and the contents of flavonoids were analyzed using HPLC-MS analysis. Five flavonoids, including f1, f2, f3, f5, and f6 as described in the previous papers were detected in 3-week-old *Arabidopsis* lines (**Figure 7A**) (Veit and Pauli, 1999; Stobiecki et al., 2006; Nakabayashi et al., 2009; Saito et al., 2013). Under UV-B irradiation, three main kinds of flavonoids (f1, f2, and f3) markedly increased in wild-type seedlings, while there was no UV-B-induced accumulation of flavonoids observed in the *uvr8* mutant seedlings (**Figures 7B–E**). In the *uvr8* mutant transformed with *CmUVR8*, UV-B-induced flavonoids accumulation was restored (**Figures 7B–E**). In addition, *CmUVR8*-overexpressing lines displayed the highest UV-B-induced flavonoids accumulation (**Figures 7B–E**). These results indicated that CmUVR8 promotes UV-B-induced flavonoids accumulation.

### The Interaction of CmUVR8 and CmCOP1

**FIGURE 8:**
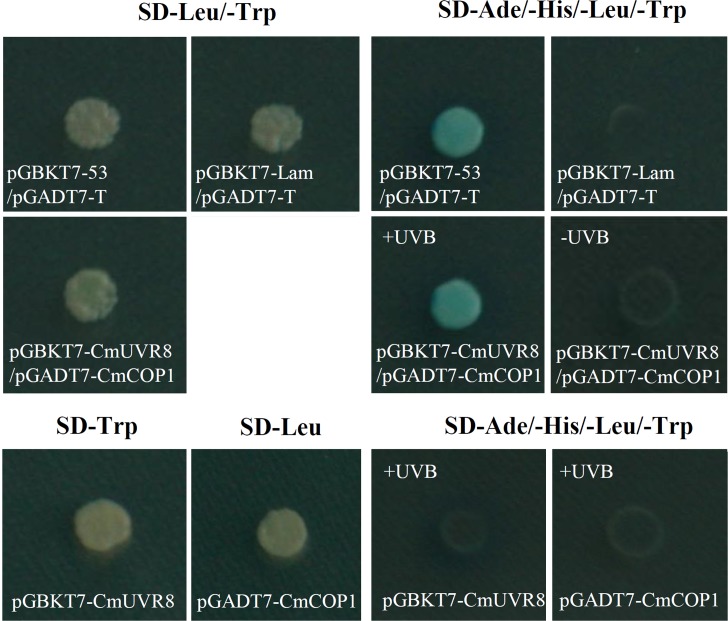
Interaction between CmUVR8 and CmCOP1. Yeast two-hybrid was performed to detect the protein interaction between CmUVR8 and CmCOP1. Yeast colonies were grown on SD-Leu/-Trp or SD-Ade/-His/-Leu/-Trp/X-α-Gal plates under 1.5 μmol m^-2^s^-1^ narrowband UV-B. Interaction of pGBKT7-53 with pGADT7-T was used as the positive control, while none interaction of pGBKT7-Lam with pGADT7-T was used as the negative control.

Although UVR8 is well-known as a long-sought-after photoreceptor, the UVR8 transduction pathway is not yet fully understood. Studies of the UVR8 photoreceptor demonstrated that the interaction between UVR8 and COP1plays important roles in UV-B perception, signal transduction and activation of the downstream UV-B specific responses in plants (Favory et al., 2009; Cloix et al., 2012). To preliminarily investigate the UVR8 signal transduction pathway in *C. morifolium*, yeast two-hybrid analysis was used to detect the interaction between CmUVR8 and CmCOP1. The full-length ORF sequences of *CmUVR8* and *CmCOP1* were cloned into the pGBKT7 and pGADT7 vectors, respectively. Both the positive control combinations and the CmUVR8-BD plus CmCOP1-AD combinations grown on SD-Leu/-Trp/-His/-Ade/X-Gal screening medium presented positive colonies (**Figure 8**). These results demonstrated that CmUVR8 interacts with CmCOP1 in *C. morifolium*.

## Discussion

UV-B is an important environmental signal perceived by plants to regulate plant growth and development. Unlike other well characterized photoreceptors, UVR8 was only recently described as a long-sought-after UV-B photoreceptor (Rizzini et al., 2011). Phylogenetic analysis showed that UVR8 proteins existed widely and are conserved in different plant species (Jenkins, 2014). In this study, *CmUVR8* gene from *C. morifolium* was isolated, and its structure and function were analyzed in detail.

### The Sequence and Structure of the CmUVR8 Protein Are Similar to AtUVR8

UVR8 is composed of a seven-bladed-propeller core, a short N-terminal extension and a flexible C-terminal region (Jenkins, 2014). UVR8 contains 14 conserved Trps, one in the C-terminus, seven in the dimer interface, and six in the β-strands (**Figure 1B**). The six Trps in the β-strands can form hydrogen bonds and hydrophobic interactions between adjacent blades, thus helping to maintain the propeller structure (O’Hara and Jenkins, 2012). Trp 285 and Trp 233 collectively serve as the UV-B chromophore (Wu et al., 2012). Although the UV-B chromophore Trps have been identified, further research is still necessary to understand how monomerization of the protein happens after UV-B photoreception. Solid evidence showed that the triad Trps (W94, W233, W285, and W337), the two Args (R286, R338) and the two Asp (D96 and D107) play important roles in maintaining the dimer structure of UVR8 (Christie et al., 2012; Wu et al., 2012; Jenkins, 2014; Heilmann et al., 2016). In addition, the three conserved GWRHT were also important for generating the triad of closely packed Trps (W233, W285, and W337). The C27 domain is required for UVR8 activity, and COP1 activity is regulated by interaction through the UVR8 C-terminal C27 domain (Yin et al., 2015).

Similar to AtUVR8, CmUVR8 protein possessed these important residues and conserved domains in its sequence, including 14 highly conserved Trps, the two Args, the two Asp, the GWRHT domain and the C27 domain (**Figure 1B**). Structure comparison of CmUVR8 and AtUVR8 showed that they have the similar 3D structures (**Figures 2A,B**). These results indicated that the function of CmUVR8 could be similar to AtUVR8.

Phylogenetic analysis of CmUVR8 and UVR8s from other plants indicated that CmUVR8 was most closely related to InUVR8, as they both belong to *Asteraceae* (**Figure 3**). In contrast, CmUVR8 was distantly related to moss and chlamydomonas as predicted (**Figure 3**).

### CmUVR8 Restored the Phenotype of Lacking UV-B-Induced Genes Expression and Flavonoids Accumulation of *uvr8* Mutant

Tissue-specific expression analysis revealed that CmUVR8 was expressed in all tissues examined, including root tissues (**Figure 4**). Under UV-B irradiation, UVR8 can first undergo monomerization of dimmers and is activated. How can this protein be activated in roots? The blue light receptor protein AtCRY1 and AtCRY2 antagonistically regulate primary root elongation through transmission of the blue-light signal from the shoot to the root by a mechanism that involves auxin (Canamero et al., 2006). Moreover, red/far-red photoreceptor proteins PHYA and PHYB can regulate both the elongation and gravitropic curvature of roots by transmission of certain hormone signals (Correll and Kiss, 2005). Apart from UV-B irradiation, there may be other signals that can activate UVR8 function in roots, such as the cryptochrome and the red/far-red photoreceptor (Correll and Kiss, 2005; Canamero et al., 2006). This hypothesis needs further research.

The high sequence similarity of CmUVR8 and AtUVR8 in conserved residues and domains suggested that CmUVR8 is the UV-B photoreceptor in *C. morifolium*. This hypothesis is verified by the functional complementation assay of CmUVR8 in *Arabidopsis uvr8* mutant (**Figure 5A**). CmUVR8 transformation restored reduced inhibition of hypocotyl elongation, and the lack of UV-B-induced increases of gene expression and flavonoids accumulation in the *uvr8* mutant (**Figures 5B,C**, **6A–E**). These experiments provided evidence that CmUVR8 is a counterpart of AtUVR8.

Previously, it was shown that quercetin glycosides were much more induced than kaempferol glycosides by UV-B under a long period of UV-B acclimation treatment (Gruber et al., 2010). In our experiments, kaempferol glycosides were much more induced by UV-B (24 h irradiation). This difference may be caused by the different UV irradiation conditions (different irradiation time and strength), as well as different age of seedlings under UV treatment.

After UV-B perception, UVR8 can interact with COP1, which links downstream UV-B-specific responses (Oravecz et al., 2006; Favory et al., 2009). This is the current known mechanism regarding UV-B response. To preliminarily explore the signaling mechanism of CmUVR8, the interaction of CmUVR8 and CmCOP1 was detected using yeast two-hybrid assays. Verification of the CmUVR8–CmCOP1 interaction indicated that the UV-B perception and signal transduction pathway in *C. morifolium* could be generally identical to *Arabidopsis*. Further research should be done to reveal the detailed UV-B signal transduction pathway in *C. morifolium*, and whether there are differences between *C. morifolium* and *Arabidopsis*.

### CmUVR8 May Play Important Roles in UV-B-Induced Accumulation of Flavonoids in *C. morifolium*

*C. morifolium* is a traditional Chinese medicine. The flower of *C. morifolium* has long been used as traditional Chinese medicine and beverage (Ye et al., 2007). Modern pharmacological studies have demonstrated that *C. morifolium* possesses extensive bioactivities and has functions of anti-inflammation (Cheon et al., 2009; Lee et al., 2009; Luyen et al., 2015), antioxidation (Lin et al., 2010; Yang, 2011), anticancer (Yuan et al., 2009; Wang et al., 2010; Yang et al., 2011; Kim et al., 2013), cardiovascular protection (Jiang et al., 2005), and hepatoprotective (Rusu et al., 2005; Gao et al., 2016). Component analysis of *C. morifolium* showed that flavonoids are the main bioactive compounds, such as luteolin-7-*O*-glucoside, quercetin-7-*O*-galactoside, apigenin-7-*O*-glucoside, diosmetin-7-*O*-glucoside, and acacetin-7-*O*-galactoside (Lin and Harnly, 2010; Sun et al., 2010).

Flavonoids have long been considered to be classical UV-B-regulated compounds in plants, and the investigation of their biosynthesis and regulation are very thoroughly (Shirley, 1996; Logemann et al., 2000). As a key enzyme in the flavonoid biosynthetic pathway, CHS has long been considered to be UV-B inducible. Although the UV-B induced accumulation of flavonoids in medicinal plants is generally acknowledged, the regulation mechanism is still largely unknown. Whether and how the newly defined UV-B photoreceptor and its signal transduction take part in this process in medicinal plants? This is an interesting question. The complementation assays of CmUVR8 suggested that CmUVR8 transformation restored the lack of UV-B-induced increases of the four important flavonoid biosynthesis genes, as well as the UV-B-induced accumulation of flavonoids in the *uvr8* mutant (**Figures 6**, **7**). These results indicated that the UV-B photoreceptor CmUVR8 probably take part in the process of UV-B-induced accumulation of flavonoids in medicinal plants.

Generally, the composition of flavonoids in medicinal plants and non-medicinal plants are very different. For example, the flavonoids in *Arabidopsis* are kaempferol glycosides and quercetin glycosides (Veit and Pauli, 1999; Stobiecki et al., 2006; Nakabayashi et al., 2009), while the main kinds of flavonoids in *C. morifolium* include luteolin-7-*O*-glucoside, quercetin-7-*O*-galactoside, apigenin-7-*O*-glucoside, diosmetin-7-*O*-glucoside, and acacetin-7-*O*-galactoside (Lin and Harnly, 2010; Sun et al., 2010). The completely different flavonoid composition indicates different metabolic pathways in the two plants. In *Arabidopsis*, the biosynthesis pathways of flavonoids are relatively clear. The major flavonoid biosynthesis enzymes are CHS, CHI, F3H, and FLS1 (Saito et al., 2013). Several flavonoid synthesis regulation genes have been screened, and some transcription factors like HY5, MYB11, MYB12, and MYB111 have been reported to be involved in the UVR8 signaling pathway (Brown et al., 2005; Stracke et al., 2010). In *C. morifolium*, the flavonoid biosynthesis genes and their regulatory genes are different from those in *Arabidopsis*. Thus, the downstream regulatory mechanisms of the UVR8 signaling pathway in *C. morifolium* should be different from those in *Arabidopsis*, although the initial regulatory mechanisms of the UVR8 signaling pathway (the regulatory relationships between UVR8, HY5, and COP1) may be similar. Further research is needed to reveal in more detail the UV-B signal transduction pathway involved in UV-B-induced accumulation of active components in medicinal plants.

## Author Contributions

MX and YY conceived and designed the research. XY, ZJ, YW, and XS performed the experiments. ZC, XR, and WJ analyzed the data. MX and YY contributed to writing the manuscript. All authors read and approved the final manuscript.

## Conflict of Interest Statement

The authors declare that the research was conducted in the absence of any commercial or financial relationships that could be construed as a potential conflict of interest.
